# Identification of a novel mechanism for reversal of doxorubicin-induced chemotherapy resistance by TXNIP in triple-negative breast cancer via promoting reactive oxygen-mediated DNA damage

**DOI:** 10.1038/s41419-022-04783-z

**Published:** 2022-04-12

**Authors:** Yiting Chen, Xueping Feng, Yuhao Yuan, Jiahui Jiang, Peihe Zhang, Bin Zhang

**Affiliations:** 1 Department of Histology and Embryology, Xiangya School of Medicine, Central South University, Changsha, 410013 China; 2 Department of Oncology and Institute of Medical Sciences, National Clinical Research Center for Geriatric Disorders, Xiangya Hospital, Central South University, Changsha, 410008 China; 3 Department of Orthopaedics, Xiangya Hospital, Central South University, Changsha, 410008 China

**Keywords:** Breast cancer, Cancer therapeutic resistance

## Abstract

Given that triple-negative breast cancer (TNBC) lacks specific receptors (estrogen and progesterone receptors and human epidermal growth factor receptor 2) and cannot be treated with endocrine therapy, chemotherapy has remained the mainstay of treatment. Drug resistance is reportedly the main obstacle to the clinical use of doxorubicin (DOX) in this patient population. Accordingly, screening molecules related to chemoresistance and studying their specific mechanisms has clinical significance for improving the efficacy of chemotherapy in TNBC patients. Thioredoxin-interacting protein (TXNIP) is a metabolism-related protein that plays a tumor suppressor role in various malignant tumors; however, the specific role of TXNIP in tumor chemoresistance has not been reported. In the present study, we explored the potential molecular mechanism of TXNIP in the chemoresistance of TNBC for the first time. The results showed that TXNIP inhibited the proliferation of TNBC drug-resistant cells and promoted apoptosis in vitro and in vivo. Furthermore, TXNIP promoted the synthesis of reactive oxygen species (ROS) and the accumulation of DNA damage caused by DOX and increased γ-H2AX levels in a time and dose-dependent manner. Moreover, ROS scavenger pretreatment could block DNA damage induced by TXNIP and restore the resistance of TNBC resistant cells to DOX to a certain extent. In addition, we found that the small molecule c-Myc inhibitor 10058-F4 promoted TXNIP expression, increased ROS synthesis in cells, and could enhance the cytotoxicity of chemotherapy drugs in vitro and in vivo when combined with DOX. These results indicated that c-Myc inhibitor 10058-F4 could induce TXNIP upregulation in TNBC drug-resistant cells, and the upregulated TXNIP increased the accumulation of ROS-dependent DNA damage, thereby decreasing chemotherapy resistance of TNBC. Our findings reveal a new mechanism of mediating drug resistance and provide a new drug combination strategy to overcome DOX resistance in TNBC.

## Introduction

Triple-negative breast cancer (TNBC) is a basal-like carcinoma with negative expression for estrogen, progesterone, and HER-2 receptors [1–3]. Chemotherapy and radiotherapy are the main treatment methods for this patient population [4, 5]; however, commonly used clinical chemotherapeutic drugs such as DOX and cisplatin can lead to drug resistance, which has become a major obstacle to effective treatment [6]. Therefore, exploring the molecular mechanisms of TNBC chemoresistance have clinical significance to improve the efficacy of chemotherapy in TNBC patients.

Many gene expression changes occur during tumorigenesis. Thioredoxin-interacting protein (TXNIP) belongs to the family of α-arrestin proteins [7], which play multiple biological roles by interacting with other functional proteins [8]. In recent years, it has been found that TXNIP is abnormally expressed in various malignant tumors [9]. Transcriptome clustering research showed that TXNIP is related to tumor chemotherapy resistance [10]. However, this interesting finding has not been reported in the literature before. In the present study, we sought to explore the role of TXNIP and the mechanisms involved in TNBC chemotherapy resistance.

With the vigorous development of tumor metabolism, some scholars have found that one of the main mechanisms of clinical treatment for many tumors is to stimulate an intracellular oxidative stress state to produce excessive intracellular ROS levels, causing death signals such as DNA damage and activation of apoptosis [11], however, tumor cells can develop a set of complex and precise regulatory mechanisms (such as initiating DNA repair systems and enhancing antioxidant capacity) during treatment and hence develop resistance [12]. Thioredoxin (TRX) is a small molecule protein that has been reported to be essential to maintain the cell’s redox balance. TRX can reduce the disulfide bond between cysteine residues in the protein, causing it to be oxidized and inactivated [13]. TXNIP is the only protein in the α-arrestin family that can bind to Trx [14] and inhibit the antioxidant function of Trx, promoting ROS accumulation. It has been established that after DNA double-strand breaks (DSBs) are induced by ionizing radiation or chemotherapeutic drugs, phosphorylation of the serine-139 residue on the C-terminal of histone H2AX located near the breakpoint occurs to form γ-H2AX. Given that phosphorylated γ-H2AX can quickly transduce DNA damage signals and trigger a series of biological cascade reactions [15], and γ-H2AX is a sensitive marker to assess DNA damage [16, 17]. Therefore, the present study explored whether the TXNIP-ROS-γ-H2AX axis mediated TNBC chemoresistance by regulating ROS-dependent DNA damage.

Interestingly, we found that the transcription factor c-Myc is the upstream negative regulator of TXNIP. Prior studies showed that by competing with the transcription factor MondoA, c-Myc could directly bind to the E-box region in the TXNIP promoter to reduce TXNIP gene expression, thereby driving glucose metabolism in TNBC cells, increasing glucose uptake, promoting cell proliferation, and reducing apoptosis [18]. In addition, MYC gene amplification has been reported in invasive breast cancer and is highly correlated with metastasis and poor prognosis [19]. Jeyshka et al. found that the knockdown of c-Myc significantly inhibited the growth of drug-resistant ovarian cancer cells [20]. 10058-F4 is a novel c-Myc small molecule inhibitor, which can reportedly disrupt the transcriptional activity of c-Myc [21]. Ghaffarnia et al. documented that 10058-F4 exerted an antitumor effect on ovarian cancer cells [22]. It remains unknown whether 10058-F4 can affect the occurrence and development of drug resistance in TNBC cells by acting on TXNIP and its downstream mechanism. Accordingly, based on the findings in the literature, we investigated the role of 10058-F4 in TNBC resistance to help in the clinical translation of the results of this study.

## Materials and methods

### Cell culture

The triple-negative breast cancer cell line MDA-MB-231 and its drug-resistant cell line MDA-MB-231/ADR were obtained from the cell bank of Xiangya Hospital of Central South University. The cells were cultured in a DMEM medium (Biological Industries, Israel) with 10% FBS (Biological Industries, Israel) and 1% penicillin/streptomycin (Gibco, USA) in a 37 °C incubator containing 5% CO_2_.

### TNBC tissue samples

The formalin-fixed paraffin-embedded (FFPE) samples of 108 patients with TNBC (between February 2014 and November 2019) were collected from Xiangya Hospital of Central South University (Hunan, China). These include doxorubicin-resistant tumor tissues and sensitive tissues, and the inclusion criteria of drug resistance in this study are as follows: 1. After neoadjuvant chemotherapy, B-ultrasound or MRI showed that the tumor volume increased or did not change; 2. The pathological Miller&Payne grading system of radical surgical specimens was classified into grade 1 and grade 2; 3. RECIST 1.1 (response evaluation criteria in solid tumors) efficacy evaluation criteria for solid tumors were defined as patients with disease progression (PD). If one of the above criteria is met, we would recognize it as chemoresistance.

We also collected detailed clinical staging and pathological data of these patients in the medical record system of Xiangya Hospital (Table 1), and anonymized them before data processing. Ethics committee approval was obtained from the ethics committee of Xiangya Hospital of Central South University, and informed consent was obtained from all patients and their legal guardians before biopsy surgery.

**Table 1:** Correlation of TXNIP expression with TNBC clinicopathologic characteristics.

		TXNIP		
Clinicopathological variable	*n*	High expression	Low expression	*P*-value
Gender				
Female	107	54	53	0.324
Male	1	1	0	
Age				
<50	79	39	40	0.593
≥50	29	16	13	
N stage				
N0	29	17	12	0.328
N1	49	25	24	
N2	20	8	12	
N3	10	5	5	
Tumor nodule number				
Solitary	51	40	11	<0.0001
Multiple (>2)	57	15	42	
Ki-67				
≥15%	69	22	47	<0.0001
<15%	39	33	6	
Distant metastasis				
Absence	43	29	14	0.005
Presence	65	26	39	

### Immunohistochemistry (IHC)

The TNBC tissue sections were deparaffinized in xylene, then rehydrated in a concentration gradient of alcohol. Subsequently, antigen retrieval was performed, and endogenous peroxidase activity was blocked with a 3% hydrogen peroxide solution. Then the TNBC tissue sections were incubated with a primary antibody (TXNIP, 1:2000, Proteintech, USA) overnight at 4 °C. The second day, after incubation with a secondary antibody (Zhongshan Golden Bridge Bio-technology, Beijing), DAB staining (Solarbio, Beijing) was performed; the cell nucleus was stained with hematoxylin slides were dehydrated and mounted on microscope slides for observation. TXNIP is mainly expressed in the cytoplasm of tumor cells. The cytoplasm staining fraction (CF) was scored as follows: 0 (0–10%), 1 (11–25%), 2 (25–50%), 3 (51–75%), or 4 (>75%). Cytoplasm staining intensity (CI) was represented as 0 (negative), 1 (weak), 2 (moderate), or 3 (strong). Then, a combined Cytoplasm Score (CS) was calculated by multiplying CF and CI (range 0–12). For statistical analyses, the cut-off values for TXNIP expression were chosen on the basis of heterogeneity using the log-rank test for OS. The optimal cut-off value was determined as low (score ≤4) or high (score >4) TXNIP expression.

### Western blot analysis

Total protein was extracted using a TPEB buffer (Invitrogen, Carlsbad, CA); the protein concentration was determined using a BCA analysis kit (Beyotime Biotechnology, Shanghai, China). The protein samples were separated by 10%/12% SDS-PAGE and then transferred to a 0.25 µm PVDF membrane (Millipore, Bedford, MA). Then the membrane was blocked in TBST containing 5% skimmed milk powder for 1 h, and incubated with the primary antibody (TXNIP, Abcam, UK; γ-H2AX, CST, USA) overnight at 4 °C. The next day, after incubation with the secondary antibody (Transgon, China) for 1 h, the membrane was washed in TBST. Finally, bands were processed using an enhanced chemiluminescence (ECL) kit (Biosharp, China).

### qRT-RCR

Total RNA was extracted using TRIzol reagent (Invitrogen, Carlsbad, CA), and reverse transcription was performed using a reverse transcription kit (Takara, Japan); then, the cDNA was amplified, according to the manufacturer’s instructions. The primer sequences required for amplification were as follows: TXNIP forward: CAGCAGTGCAAACAGACTTCGG, reverse: CTGAGGAAGCTCAAAGCCGAAC; GAPDH forward: GTCTCCTCTGACTTCAACAGCG, reverse: ACCACCCTGTTGCTGTAGCCAA.

### Construction of cell lines stably expressing TXNIP

The overexpression lentivirus OE-TXNIP, shRNA1, and shRNA2 for TXNIP, and their corresponding control lentivirus Con313 (hU6-MCS-CBh-gcGFP-IRES-puromycin) and Con335 (CMV-enhancer-MCS-3FLAG-EF1-Z-sGreen1-T2A-puromycin) were synthesized by the company Gikai GENE. Stable cell lines were screened with 2 µg/mL puromycin.

### Immunofluorescence (IF) staining and confocal microscopy analysis

The cells were inoculated in a 12-well plate with nest climbing tablets in advance. After treatment with DOX for 48 h (optional), the cells were washed three times and fixed with 4% paraformaldehyde for 10 min. The cells were gently washed with PBS twice, permeabilized with 0.1% Triton X-100 (Solarbio, Beijing) at room temperature for 10 min, blocked with 3% BSA for 30 min, and finally incubated with the primary antibody (diluted in the above 3% BSA) overnight (be careful to keep in a humid box for moisture). The next day, the cells were washed with PBS for 30 min, and PBS was replaced every 5 min. Subsequently, the cells were incubated with fluorescent secondary antibody for 1 h (protected from light), washed with PBS for 30 min, and dyed with DAPI (Servicebio, Wuhan, China) for 5 min. The climbing slide was buckled on the slide, sealed with glycerol, observed, and photographed under the laser confocal microscope. After all pathological sections were deparaffinized and rehydrated, antigen retrieval was performed. After blocking with 3% BSA solution for 30 min, the tissue sections were incubated with primary antibody overnight at 4°. The subsequent steps were consistent with the principle of cellular immunofluorescence.

### Colony formation assay

The cells were seeded in a 6-well culture plate at a 600 cells/well density and cultured with DMEM complete medium for 2 weeks. After being fixed with 4% paraformaldehyde for 20 min and stained with crystal violet for 15 min, photographs were taken with a camera, and the number of colonies was calculated with Image J software.

### 5-ethynyl-2′-deoxyuridine (EdU) staining

The cells were seeded in a 96-well plate (2 × 10^4^ cells per well), and cell proliferation was measured with the EdU (5-ethynyl-2′-deoxyuridine) kit (RiboBio, China). EdU is a thymidine analog that infiltrates thymine (T) into the synthesized DNA during DNA replication. The results were observed under a fluorescence microscope.

### Cell viability assay

A CCK-8 kit (Biosharp, China) was used to evaluate the cell viability, according to the manufacturer’s instructions. The cells were inoculated into 96-well plates. After the cells adhered to the walls of the culture plate, drugs of different concentration gradients were added to each well for 24/48 h, then the CCK-8 reagent was added into 96-well plates (10 µL/well) and incubated at 37 °C for 2 h. The absorbance was measured at 450 nm. Cell viability was calculated by the formula (cell viability (%) = [A (dosing) − A (blank)]/[A (0 dosing) − A (blank)] × 100).

### Cell apoptosis assay

The cell suspension and cells digested with EDTA-free trypsin were collected and washed twice with pre-cooled PBS, then stained using an apoptosis detection kit (Vazyme, China). After incubation at room temperature in the dark for 10 min, the stained samples were analyzed by flow cytometry within 1 h.

### Reactive oxygen species (ROS) analysis

The digested cells and cell suspension were placed in a centrifuge tube, DHE (US Everbright, Suzhou) and diluted with serum-free and antibiotic-free DMEM medium (ratio 1:1000), according to the manufacturer’s instructions, to obtain a final DHE concentration of 10 μM. The cells were suspended in 500 μl of the diluted DHE and cultured in an incubator at 37 °C for 20 min. Then, the cells were washed twice with serum-free and antibiotic-free DMEM. The fluorescence intensity of DHE was detected by flow cytometry, which reflected the intracellular ROS levels. For adherent cells, after DHE incubation and serum-free DMEM cleaning, the cell climbing slides were buckled on the slide, and ROS expression was observed under a confocal microscope.

### Xenograft mouse models

The animal experiments in this study were approved by the Animal Care and Ethics Committee of Xiangya Hospital of Central South University (Changsha, China). Four- to six-week-old female nude mice (BALB/C) were divided into three groups consisting of 231/ADR cells stably transfected with OE-TXNIP, MDA-MB-231 cells, and 231/ADR cells stably transfected with si-TXNIP (4 × 10^6^ cells/mouse) injected into the right upper back of nude mice. The tumor volume of mice was measured every 3 days. When tumor tissue was visible to the naked eye under the back of nude mice, the c-Myc inhibitor 10058-F4 (20 mg/kg, MedChemExpress, Shanghai) and DOX (10 mg/kg, Solarbio, Beijing) were injected intraperitoneally. The injection was repeated every 3 days. When the tumor tissue under the skin of nude mice grew for 3 weeks, the nude mice were anesthetized and euthanized, and the tumor size was measured with a caliper.

### Data analysis

SPSS version 20 (SPSS Inc, Chicago, IL, USA) and GraphPad Prism 8 were used for data processing and statistical analysis of the experimental results. The student’s *t*-test was used for the inter-group comparison of quantitative data, while analysis of Chi-square test or Fisher’s exact test was used for the inter-group comparison of qualitative data expressed in frequency. A *p*-value < 0.05 was statistically significant.

## Results

### Association of TXNIP expression with TNBC clinicopathological characteristics

The relationship between low or high TXNIP expression and typical clinicopathological parameters is shown in Table 1. Among the 108 primary TNBC tissues, 49.1% (53/108) had low TXNIP expression (IHC score ≤4), and 50.9% (55/108) had high TXNIP expression (IHC score >4). As shown in Table 1, TXNIP expression was not significantly correlated with gender, age, and breast cancer stage (N), but was negatively correlated with Ki-67 expression, tumor nodule number, and distant metastasis. These results suggest that decreased expression of TXNIP may be involved in the progression of TNBC.

### TXNIP was lowly expressed in TNBC drug-resistant tissues and cells

**Fig. 1:**
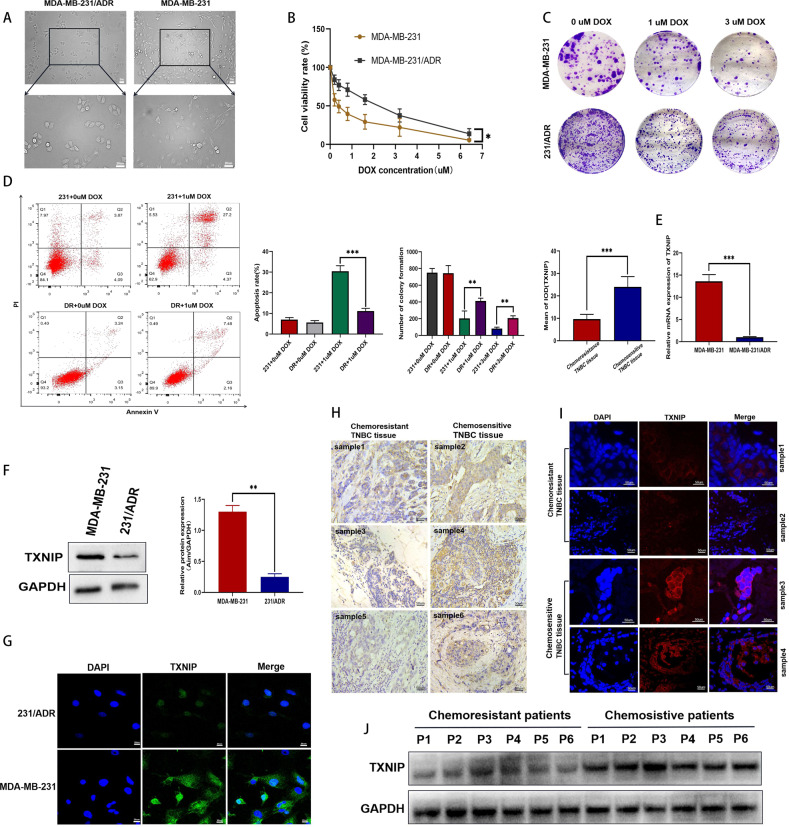
Low expression of TXNIP in TNBC drug-resistant tissues and cells. **A** The morphological differences between 231/ADR cells and 231 cells under a light microscope. **B** Analysis of DOX toxicity levels in 231/ADR cells and 231 cells. **C** Under the same DOX concentration, the clonogenic ability of 231/ADR cells was stronger than that of 231 cells. **D** Under the same DOX concentration, the apoptotic rate of 231 cells was greater than that of 231/ADR cells. **E** At the mRNA level, TXNIP expression in 231/ADR cells was significantly lower than in 231 cells. **F** WB experiment confirmed low expression of TXNIP in 231/ADR cells. **G** Immunofluorescence showed a difference in TXNIP expression between 231 cells and 231/ADR cells. **H** Immunohistochemistry showed that TXNIP was highly expressed in the tissues of chemotherapy-sensitive patients (samples 1–6 are different cases). Bar: 50 μm. **I** Tissue immunofluorescence shows low TXNIP expression in TNBC tissues from chemotherapy-resistant patients (samples 1–4 are different cases). **J** The expression difference of TXNIP in patient tissues (P1–P6). Bar: 25 μm or 50 μm. Data were shown as the mean ± SD from three independent experiments. *P < 0.05; **P < 0.01; ***P < 0.001.

Subsequently, we observed under an ordinary microscope that the TNBC sensitive cell line MDA-MB-231 has a larger morphology than the drug-resistant MDA-MB-231/ADR and has more pseudopods, while the 231/ADR morphology is generally like round (Fig. 1A). The CCK-8 assay showed that IC50 values of the sensitive and resistant strain were 0.36 μM and 1.86 μM, respectively (Fig. 1B). After adding different concentrations of DOX, 231/ADR cells exhibited stronger colony-forming ability than 231 cells at the same drug concentration (Fig. 1C). The apoptotic ratio of 231 cells was much higher than that of 231/ADR cells (Fig. 1D), indicating the significantly lower sensitivity of 231/ADR cells to DOX, to reach the standard of our subsequent experiments. Subsequently, we analyzed the difference in TXNIP expression in both cell lines. Western blot showed that TXNIP protein levels in 231/ADR were significantly lower than in 231 cells (Fig. 1F). qRT-PCR also confirmed significantly lower TXNIP mRNA levels in 231/ADR cells than in 231 cells (Fig. 1E), while the cell immunofluorescence results validated low TXNIP expression in 231/ADR cells (Fig. 1G). Pathological tissue sections of TNBC were analyzed by immunohistochemistry and tissue immunofluorescence. It was found that TXNIP was highly expressed in TNBC tissues of patients sensitive to chemotherapy, and low expression was found in tissues from patients resistant to chemotherapy (Fig. 1H, I). We also extracted tissue proteins, and through WB detection, it was found that TXNIP was highly expressed in patients who were sensitive to chemotherapy, but was low in patients who were resistant to chemotherapy (Fig. 1J).

### TXNIP expression was induced by DOX in TNBC drug-sensitive and drug-resistant cells

**Fig. 2:**
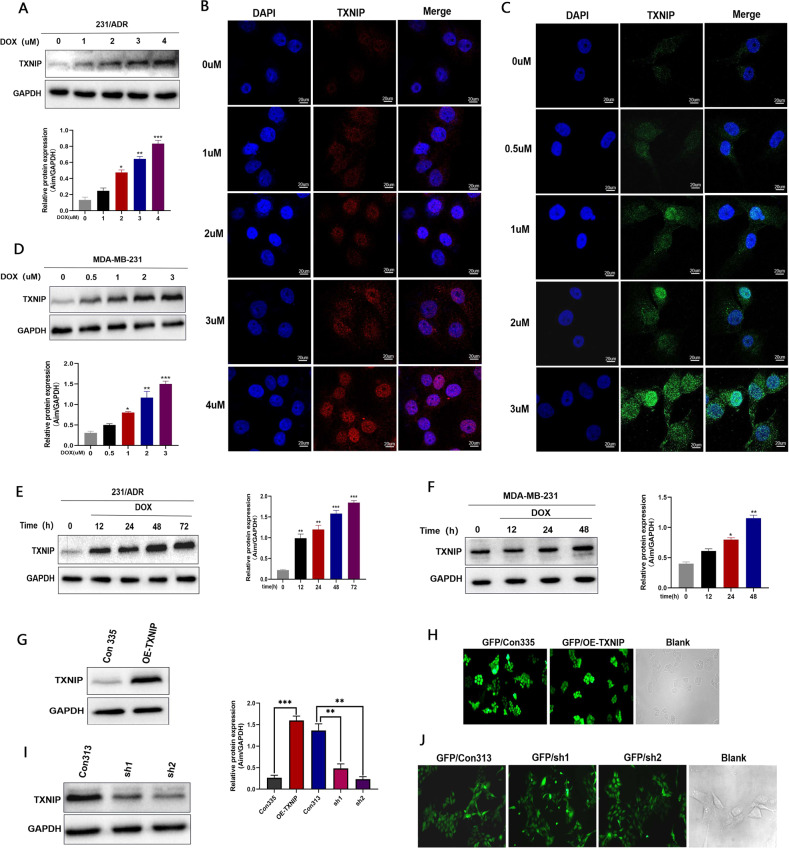
DOX-induced TXNIP expression in 231 cells and 231/ADR cells. **A**, **D** 231/ADR cells and 231 cells were treated with different concentrations of DOX for 48 h. **B** The immunofluorescence assay showed that TXNIP was upregulated with the drug concentration gradient in 231/ADR cells. **C** Immunofluorescence showed that TXNIP was upregulated under a drug concentration gradient in 231 cells. **E** 231/ADR cells were treated with DOX (2 μM) and collected at the indicated time. **F** 231 cells were treated with DOX (1 μM) and collected at the indicated time. **G** A stable TXNIP overexpression cell line cells were established in 231/ADR. **H**, **J** The transfection efficiency of overexpression and knockdown under a fluorescence microscope. **I** A stable TXNIP knockdown cell line was established in 231 cells. Data were presented as mean ± SD of three independent experiments. *P < 0.05, **P < 0.01; ***P < 0.001.

To investigate whether TXNIP plays a role in chemotherapy resistance in TNBC, we analyzed the effect of DOX on TXNIP expression in both cell lines. WB results showed that DOX upregulated TXNIP expression in both cell lines in a dose-dependent (Fig. 2A, D) and time-dependent (Fig. 2E, F) manner. Immunofluorescence results also showed that TXNIP expression was positively correlated with DOX drug concentration in 231/ADR (Fig. 2B) and 231 (Fig. 2C) cell lines, indicating that doxorubicin upregulated TXNIP expression at the protein level.

### TXNIP reverses DOX-induced chemotherapy resistance in TNBC

**Fig. 3:**
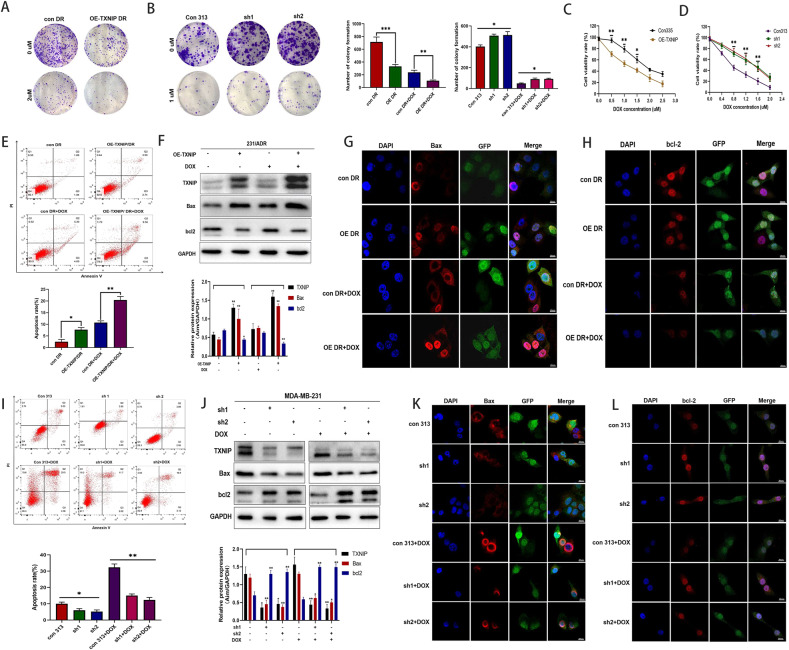
TXNIP decreased doxorubicin-induced chemotherapy resistance in TNBC. **A**, **B** Colony-formation was visualized on day 14. **C**, **D** Changes in the sensitivity of 231/ADR cells and 231 cells to DOX after overexpression of TXNIP and knockdown of TXNIP. **E**, **I** Apoptotic cells were analyzed by flow cytometry. **F**, **J** Expression changes of Bax and bcl-2 protein in 231/ADR and 231 cells. **G**, **H** Immunofluorescence of Bax and bcl-2 in 231/ADR cells transfected with OE-TXNIP shRNA plasmid. **K**, **L** Immunofluorescence of Bax and bcl-2 in 231 cells transfected with TXNIP shRNA plasmid. Bar: 10 μm. Data were presented as mean ± SD of three independent experiments. *P < 0.05; **P < 0.01; ***P < 0.001.

To investigate the effect of TXNIP expression on the efficacy of chemotherapeutic agents, cell lines stably overexpressing TXNIP (OE-TXNIP) were constructed in 231/ADR cells, and transfection efficiency was confirmed by WB (Fig. 2G). Meanwhile, GFP fluorescence that comes with lentivirus was also observed under a confocal microscope (Fig. 2H). In addition, cell lines stably knockdown TXNIP (sh-TXNIP) were constructed in 231 cells, knockdown efficiency was confirmed by WB (Fig. 2I), and significant GFP fluorescence was observed under a confocal microscope (Fig. 2J). Next, we examined the effect of TXNIP on TNBC proliferation by the colony formation assay; the results showed that TXNIP overexpression inhibited the colony formation ability of 231/ADR, with a more significant inhibition effect after drug administration (Fig. 3A), while knockdown of TXNIP promoted the colony formation ability of 231 cells (Fig. 3B), Then, CCK-8 was used to detect the DOX toxicity levels on stable cells in each group. The results showed that TXNIP overexpression significantly increased the sensitivity of 231/ADR cells to chemotherapy (Fig. 3C) while knocking down TXNIP significantly reduced the sensitivity of the 231 cells to chemotherapy (Fig. 3D). In addition, the results of flow cytometric detection of apoptosis showed that TXNIP exacerbated the apoptosis of 231/ADR cells induced by DOX (Fig. 3E), while the WB assay demonstrated that the expression of anti-apoptotic protein bcl-2 and pro-apoptotic protein Bax was downregulated and upregulated, respectively (Fig. 3F). Similarly, after knocking down TXNIP, the apoptotic rate of 231 cells induced by DOX was significantly reduced (Fig. 3I), while expression of bcl-2 and Bax was significantly upregulated and downregulated, respectively (Fig. 3J). The results of immunofluorescence assay also found that TXNIP overexpression in 231/ADR cells enhanced Bax expression shown as the red particles (Fig. 3G) and decreased the expression of bcl-2 (Fig. 3H), while knockdown of TXNIP in 231 cells reduced the expression of Bax (Fig. 3K) and increased the expression of bcl-2 (Fig. 3L).

### TXNIP overexpression decreased resistance to DOX in vivo

**Fig. 4:**
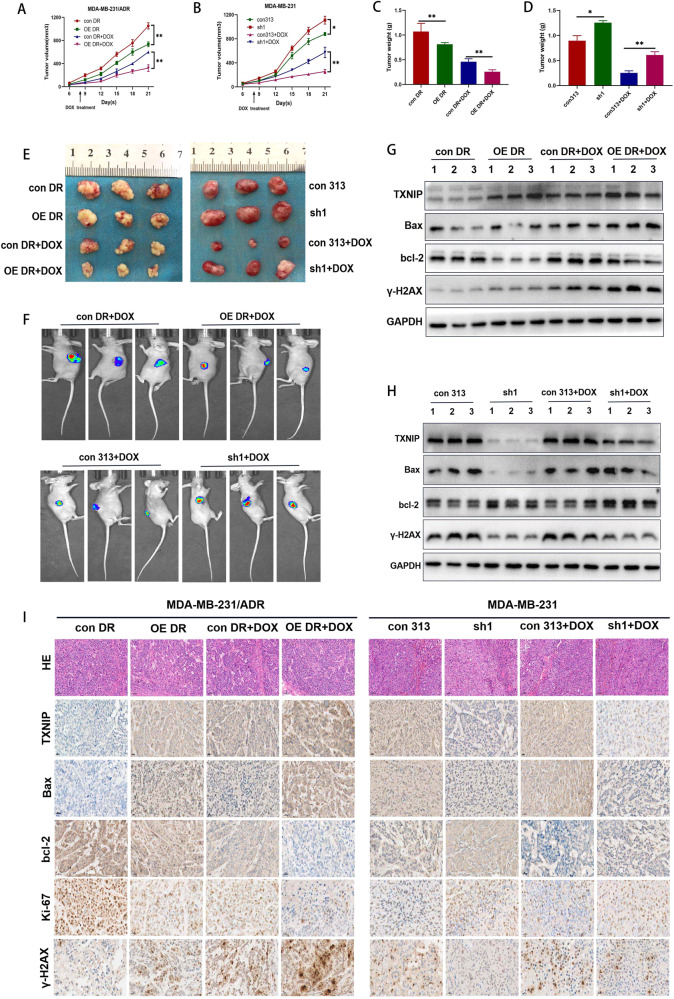
TXNIP mediates TNBC sensitivity to DOX in vivo. **A**, **B** Tumor growth was examined by measuring the tumor volume after 2 weeks of treatment with DOX (10 mg/kg, every 3 days, *n* = 6); the average tumor volumes in each group were calculated using the following formula: volume = (*a* × *b*^2^)/2, where *a* represents the long diameter, and *b* represents the short diameter (both *a* and *b* were measured using a Vernier caliper). **C**, **D**, **E** Three weeks after subcutaneous implantation, the tumor was peeled off and weighed. **F** Two weeks after subcutaneous transplantation, the difference in tumor growth was visually observed under live-animal imaging. **G**, **H** The expression difference of TXNIP, bcl-2, Bax, γ-H2AX in transplanted tumors. **I** Immunohistochemistry staining for apoptosis-related protein, TXNIP, γ-H2AX, and Ki67 expression in subcutaneous tumors (original magnification ×400). Data are shown as the mean ± SD from three independent experiments. *P < 0.05, **P < 0.01; ***P < 0.001.

To investigate the potential effect of TXNIP on enhancing sensitivity to DOX in vivo, 231 and 231/ADR cells with different TXNIP expression levels were subcutaneously injected into nude mice treated with or without DOX. 231/ADR and 231 tumors that exhibited high TXNIP expression grew at a significantly lower rate than those with lower expression (Fig. 4A, B). On day 21 after inoculation, the tumor was removed and tumor weight was measured (Fig. 4C, D). Furthermore, the volume of 231/ADR xenografts in the OE-TXNIP DOX group was smaller than in the con DR DOX group, the tumor volume of the 231 xenografts in the sh-TXNIP DOX group was larger than that of the con313 DOX group (Fig. 4E). Live-animal imaging validated the above results (Fig. 4F). Protein was extracted from the tumors of the three groups of mice. Western blot analysis showed expression differences of TXNIP, Bax, bcl-2, γ-H2AX in transplanted tumors of nude mice(Fig. 4G, H). In addition, higher Bax, TXNIP, and γ-H2AX, and lower bcl-2 and Ki67 expression levels were observed in the TXNIP-high compared to the con DR group (Fig. 4I). These data were consistent with those obtained in vitro.

### TXNIP induced ROS overproduction and enhanced DOX-induced DNA damage

**Fig. 5:**
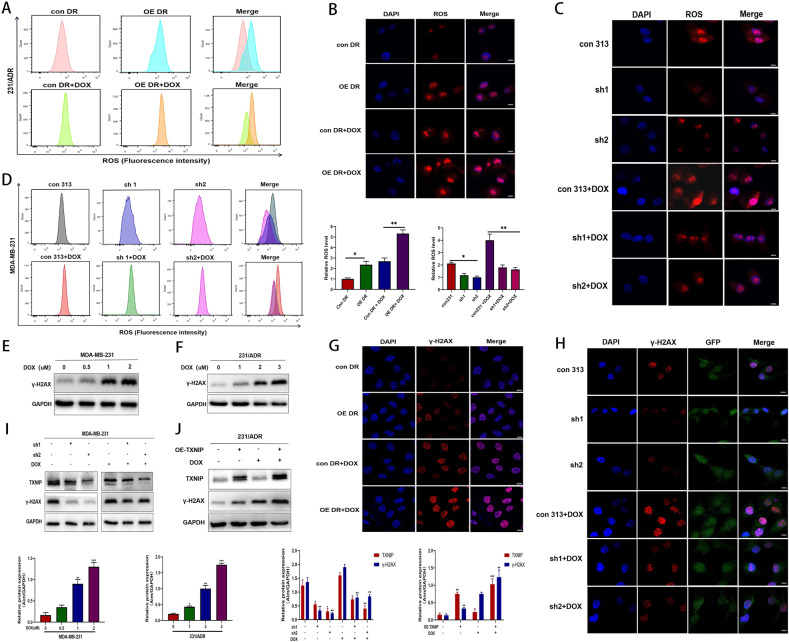
TXNIP induced ROS overproduction and enhanced doxorubicin-induced DNA damage. **A**, **D** 231/ADR and 231 intracellular ROS levels were determined by flow cytometry. **B**, **C** Representative images of ROS synthesis in 231/ADR and 231 cells. Quantification of relative ROS level in tested cells. Data are represented as mean ± SD from three independent experiments performed in triplicate. **E**, **F** The expression level of γ-H2AX protein under a series of concentration gradient DOX solutions in 231 and 231/ADR cells. **G**, **H** The representative images of immunofluorescence of γ-H2AX in 231/ADR and 231 cells transfected with OE-TXNIP or sh-TXNIP. **I**, **J** The expression level of γ-H2AX in 231/ADR and 231 cells transfected with OE-TXNIP or sh-TXNIP was detected by WB. Bar: 10 μm. Data are presented as mean ± SD of three independent experiments. *P < 0.05; **P < 0.01; ***P < 0.001.

To clarify the role of TXNIP in the regulation of DOX resistance, we used a DHE probe to analyze the intracellular ROS levels. Flow cytometry showed that TXNIP upregulation increased DOX-induced ROS levels in 231/ADR cells (Fig. 5A), while TXNIP downregulation reduced ROS levels in 231 cells (Fig. 5D). Subsequently, we loaded DHE probes into the adherent 231/ADR and 231 cells. Under confocal microscopy, we found that TXNIP overexpression enhanced the elliptical mass red fluorescence in 231/ADR cells (Fig. 5B) while knocking down TXNIP weakened the red fluorescence in 231 cells (Fig. 5C). The above results showed that TXNIP could promote DOX-induced ROS synthesis in 231/ADR and 231 cells. Next, we studied the effect of TXNIP induced DNA damage. γ-H2AX is widely acknowledged as a DNA double-strand break marker. The WB assay showed that a DOX concentration gradient could upregulate γ-H2AX protein levels in 231 cells (Fig. 5E) and 231/ADR cells (Fig. 5F). TXNIP downregulation significantly reduced γ-H2AX expression induced by DOX in 231 cells (Fig. 5I). In contrast, TXNIP overexpression significantly increased γ-H2AX expression in 231/ADR cells, especially after treatment with DOX. Furthermore, TXNIP upregulation led to more significant DNA damage in 231/ADR cells (Fig. 5J). In addition, immunofluorescence results showed that TXNIP overexpression could induce the formation of γ-H2AX lesions in 231/ADR cells under DOX treatment (Fig. 5G), and knockdown of TXNIP just reduced the incidence of DAPI andγ-H2AX lesions co-staining in 231 cells (Fig. 5H). Accordingly, the above findings suggest that TXNIP can enhance DNA damage induced by DOX.

### ROS scavenger DMTU blocked TXNIP-induced DNA damage and partially restored the resistance of TNBC resistant cells to DOX

**Fig. 6:**
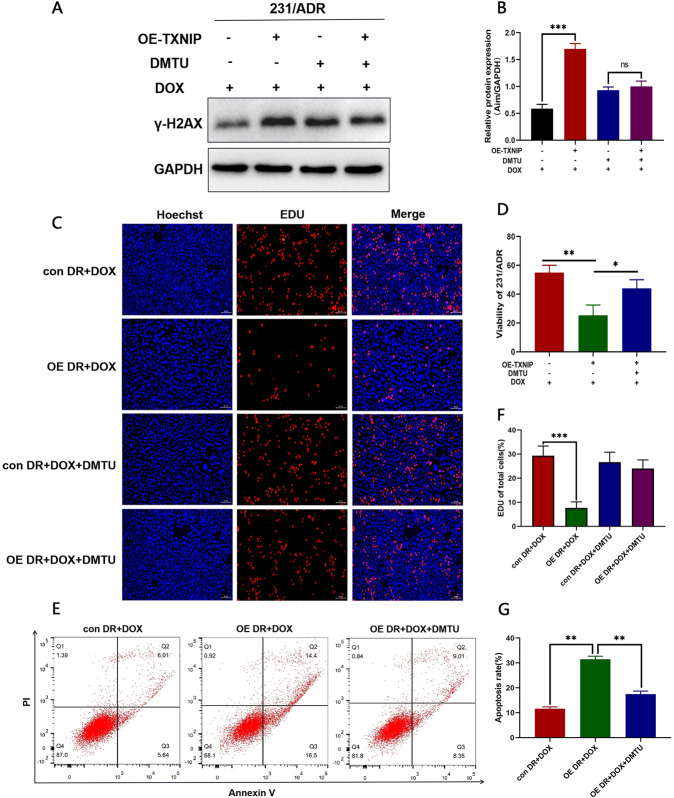
DMTU partially restored the resistance of 231/ADR to doxorubicin. **A**, **B** 231/ADR cells were treated with DMTU, andγ-H2AX were detected by western blot. **C**, **F** The proliferation capacity of 231/ADR was measured by the EdU assay after being treated with DOX and DMTU. **D** 231/ADR cells were treated with DOX for 24 h in the presence or absence of DMTU, cell viability was determined by CCK8 assay. **E**, **G** Flow cytometric detection of apoptosis in 231/ADR after being treated with DOX for 48 h in the presence or absence of DMTU. Bar: 10 μm. Data are presented as mean ± SD of three independent experiments. *P < 0.05; **P < 0.01, ***P < 0.001, ns = no significance.

To assess whether ROS levels drive the activation of the DNA damage response in drug-resistant TNBC cells, we used DMTU (N,N’-Dimethylthiourea), a ROS scavenger. As shown in Fig. 6A and B, after using DMTU, γ-H2AX expression in the 231/ADR cells that TXNIP initially upregulated was downregulated to a certain extent. The EdU assay (Fig. 6C, F) confirmed that DMTU could partially restore the proliferation ability of cells inhibited by TXNIP. Moreover, the CCK-8 assay (Fig. 6D) demonstrated that the intracellular signaling events induced by TXNIP were ROS-dependent. Flow cytometry showed that the addition of DMTU could significantly inhibit TXNIP-induced apoptosis of 231/ADR cells (Fig. 6E, G). These findings indicated that the anti-proliferative and pro-apoptotic effects of TXNIP on chemoresistant TNBC cells were partly induced by ROS-dependent DNA damage.

### The inhibitor 10058-F4 upregulated TXNIP to promote ROS level and accumulation of DNA damage, and synergised with DOX in 231/ADR cells

**Fig. 7:**
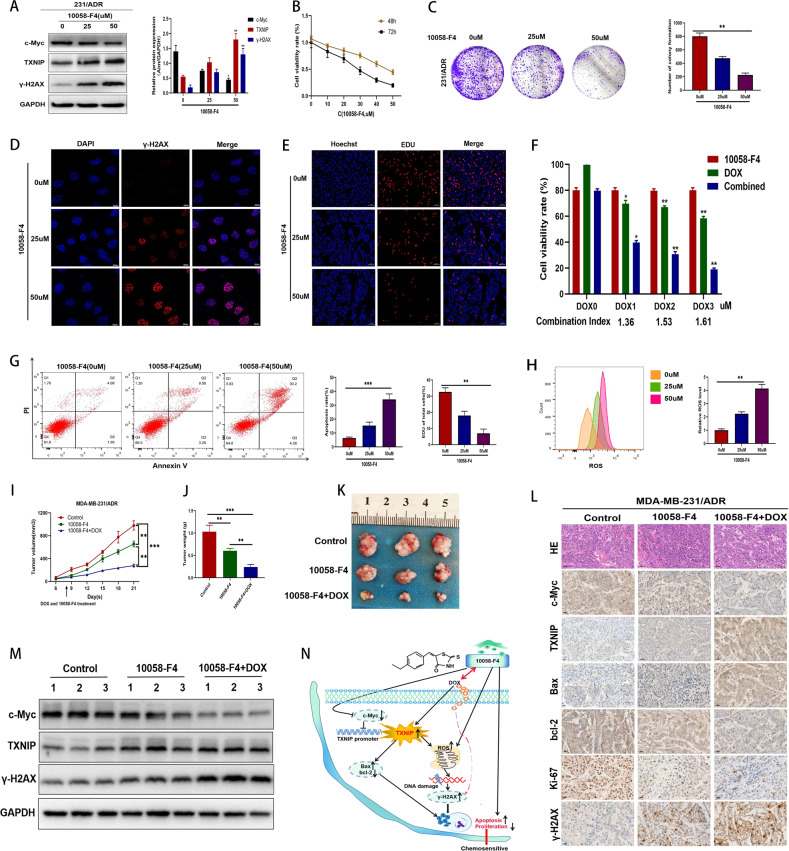
The role of 10058-F4 in TNBC chemotherapy resistance. **A** The expression levels of c-Myc, TXNIP, γ-H2AX in 231/ADR cells with different concentrations of 10058-F4 were analyzed by western blot. **B** The proliferation of 231/ADR cells treated with a 10058-F4 concentration gradient by CCK-8 (24/48 h). **C** The colony formation assay measured the clonogenic ability of 231/ADR cells after being treated with 10058-F4 for 10 days. **D** The representative images of immunofluorescence of γ-H2AX in 231/ADR treated with 10058-F4. **E** EdU detected the proliferation of 231/ADR cells under 10058-F4 treatment. **F** The CCK-8 assay measured the therapeutic effect of 10058-F4 in combination with DOX. The combination index was calculated below. **G** Apoptosis of 231/ADR cells was detected by flow cytometry under 10058-F4 treatment. **H** 231/ADR cells were exposed to different concentrations of 10058-F4 for 48 h, then treated cells were stained with DHE probe for 30 min, cellular ROS levels were determined by flow cytometry. **I**, **J**, **K** Tumor volume change curve, weight, and size after 10058-F4 and DOX were combined. **L** Immunohistochemistry staining for apoptosis-related protein, TXNIP, c-Myc, γ-H2AX, and Ki67 expression in subcutaneous tumors (original magnification ×400). **M** WB for apoptosis-related protein, TXNIP, c-Myc, γ-H2AX, bcl-2, Bax, and Ki67 expression in subcutaneous tumors. **N** In 231/ADR cells, the c-Myc inhibitor 10058-F4 upregulated TXNIP expression, which promoted ROS synthesis and the accumulation of DNA damage, thereby reversing the chemotherapy resistance of TNBC. In addition, 10058-F4 and DOX can work synergistically to enhance the sensitivity of TNBC to DOX. Data are shown as the mean ± SD from three independent experiments. *P < 0.05, **P < 0.01; ***P < 0.001.

Considering the tumor-proliferating effect of c-Myc on drug-resistant malignant tumor cells [22] and the regulatory effect of c-Myc on TXNIP. Accordingly, we used the c-Myc inhibitor 10058-F4 [23]. After treatment with the c-Myc inhibitor 10058-F4, the c-Myc level in 231/ADR cells was decreased in a dose-dependent manner, while the TXNIP and γ-H2AX levels were upregulated (Fig. 7A). In addition, immunofluorescence analysis showed that γ-H2AX lesions were significantly enhanced in the nuclei of 10058-F4 treated cells (Fig. 7D), indicating that 10058-F4 induced DNA damage in 231/ADR cells. CCK-8 and colony formation experiments showed that 10058-F4 significantly inhibited cell survival and colony formation in vitro (Fig. 7B, C). Similarly, EdU assays confirmed that 10058-F4 could inhibit the proliferation of 231/ADR cells (Fig. 7E). Subsequently, flow cytometry showed that 10058-F4 could dose-dependently promote the apoptosis of 231/ADR cells (Fig. 7G) and increase ROS levels in 231/ADR cells (Fig. 7H). Taking into account the resistance to DOX, a CCK-8 assay was performed to assess the efficacy of 10058-F4 in combination with DOX. 231/ADR cells were treated with a specific dose of 10058-F4, and different concentrations of DOX were added; the combination index was then calculated. The results demonstrated a synergistic effect (*Q* > 1.15) between 10058-F4 and DOX (Fig. 7F), which suggested that the combination of 10058-F4 and DOX may be a promising combination strategy against TNBC resistance.

### Combination therapy of 10058-F4 and DOX enhanced chemosensitivity in a xenograft mouse model

Subsequently, the in vivo effect of the combination of 10058-F4 with DOX was assessed using a murine xenograft model. The tumor growth rate of nude mice treated with the combination of 10058-F4 and DOX significantly slowed down from the 12 day of treatment (Fig. 7I). At the end of the drug treatment, the tumor size and weight of the mice receiving the combination therapy were significantly lower than those of the other groups (Fig. 7J, K), indicating the effectiveness of the drug combination. Moreover, after the drug combination treatment, we found that TXNIP, Bax, and γ-H2AX expressions were upregulated while the bcl-2 and Ki67 expressions were downregulated in the tumor sections of nude mice (Fig. 7L). In addition, the expression level of TXNIP, c-Myc, γ-H2AX were consistent with the in vitro results (Fig. 7M).

## Discussion

TNBC is characterized by the lack of ER, PR, and HER2 receptors, the key factor that ultimately determines the survival and prognosis of TNBC patients is the efficacy of chemotherapy. However, many TNBC patients are prone to drug resistance after exposure to neoadjuvant chemotherapy and eventually die from tumor recurrence or metastasis [24, 25]. Accordingly, finding new targets for decreasing drug resistance and clarifying the underlying mechanisms of action are critical to improve the prognosis for TNBC. To the best of our knowledge, this is the first study to substantiate that TXNIP could promote ROS-dependent DNA damage, thereby reversing DOX-induced chemotherapy resistance in TNBC. Furthermore, we demonstrated that the c-Myc inhibitor 10058-F4 could work synergistically with DOX to inhibit the proliferation of drug-resistant cells and promote their apoptosis in TNBC.

Previous studies have shown that TXNIP exerts a tumor suppressor effect on the occurrence and development of liver cancer, breast cancer, and lung cancer [26–29] and plays a key role in mediating apoptosis and tumor cell cycle arrest [30–32]. Hangsak Huy et al. [33] confirmed that the TLR4/NF-kB axis could induce fludarabine resistance by inhibiting TXNIP expression in acute myeloid leukemia cells. In the meantime, our research group conducted gene sequencing of early-stage non-small cell lung cancer and found that TXNIP was highly correlated with cisplatin resistance. However, it remains unknown whether TXNIP participates in TNBC chemoresistance. This study corroborated that TXNIP is a drug-sensitive gene in TNBC and can be upregulated by a DOX concentration gradient. Moreover, upregulation of TXNIP induced by DOX inhibited proliferation and promoted apoptosis of MDA-MB 231/ADR cells; however, downregulation of TXNIP promoted cell proliferation and inhibited apoptosis in 231 cells. In vivo experiments also validated that TXNIP could inhibit the formation of transplanted tumors in nude mice and decrease resistance. The subcutaneous tumor formed by 231/ADR cells was lobulated and hard, while the subcutaneous tumor formed by 231 cells was smooth and soft, suggesting that tumorigenesis in 231/ADR cells exhibited a relatively more malignant phenotype. Quantification of apoptosis-related proteins Bax, bcl-2, and proliferation-related protein Ki67 in xenograft tumor sections showed that TXNIP upregulation could promote the apoptosis of DOX-induced drug-resistant cells and inhibit their proliferation in vivo. The above findings corroborate that TXNIP reduces the resistance of DOX-resistant TNBC cells and enhances the sensitivity of DOX-sensitive TNBC cells.

Li Yan et al. [34] advocated that TXNIP is a key regulator of the cellular redox status and can inhibit the antioxidant function of thioredoxin. ROS play an antitumor role predominantly by promoting tumor cell apoptosis and tumor cell necrosis and participating in autophagy cell death [35, 36]. Li Jian et al. [37] found that TXNIP overexpression inhibits the proliferation of liver cancer cells by triggering mitochondrial-mediated ROS generation. The above studies showed that TXNIP promotes oxidative stress and can be leveraged as a potential antitumor treatment strategy. Given that DOX can increase ROS levels, this study used flow cytometry to quantify ROS synthesis. We found that TXNIP increased ROS synthesis and promoted apoptosis in 231/ADR cells, while the downregulation of TXNIP in 231 cells led to decreased ROS synthesis and cell apoptosis. In addition, TXNIP-induced apoptosis of 231/ADR cells could be inhibited by the ROS scavenger DMTU. These results suggest that ROS play an important role in TXNIP-induced apoptosis of drug-resistant cells.

A large number of studies have shown that ROS can cause oxidative damage and DNA damage [38], including DNA double-strand breaks (DSBs) [12], which can alter the sensitivity of tumor cells to chemotherapeutic drugs [39]. Interestingly, we found that the expression of γ-H2AX increased after DOX treatment in a dose- and time-dependent manner. In addition, TXNIP overexpression can lead to significant upregulation of DOX-induced γ-H2AX protein levels, which indicates that TXNIP prolongs the DSBs repair process induced by DOX. Accordingly, TXNIP-ROS-γ-H2AX axis may be a new and effective method to combat TNBC chemotherapy resistance. Next, 231/ADR cells overexpressing TXNIP were treated with the ROS scavenger DMTU. We found that γ-H2AX protein expression decreased to a certain extent. Most importantly, TXNIP overexpression led to increased apoptosis and reduced the proliferation of 231/ADR cells, which could be reversed by DMTU to a certain extent. The semiquantitative analysis found that DMTU restored cell function by more than 50%. However, it is worth noting that ROS scavenging did not completely block sensitivity to chemotherapy, indicating a high likelihood that other mechanisms are involved. Indeed, more studies are required to explore whether the effect of TXNIP on TNBC chemoresistance is mediated by autophagy, ferroptosis, metabolic reprogramming, or other processes.

Several studies have demonstrated that TXNIP is a negative transcriptional regulator of c-Myc. 10058-F4 is a small molecule inhibitor of c-Myc that has a strong antitumor effect [40, 41]. Although 10058-F4 monotherapy has achieved positive results in cell lines and preclinical models of malignant tumors such as ovarian cancer and liver cancer, there are no reports on the treatment of invasive TNBC [42] or the improvement of TNBC chemoresistance. Therefore, the study of multi-drug combinations in TNBC has significant clinical value in guiding treatment selection. To the best of our knowledge, this is the first study to demonstrate that 10058-F4 can upregulate TXNIP and γ-H2AX expression in 231/ADR cells and induce ROS synthesis. It can also inhibit proliferation and promote apoptosis of 231/ADR cells. Furthermore, 10058-F4 combined with DOX can synergistically inhibit the proliferation of 231/ADR cells. Interestingly, in the transplanted tumor model, we found that 10058-F4 can inhibit the formation of subcutaneous tumors, and tumor formation was significantly inhibited with the drug combination of 10058-F4 and DOX, suggesting that 10058-F4 as a single drug or combined with DOX has clinical value in decreasing the resistance of TNBC cells to DOX. However, some limitations were present in our study since it remains unclear whether the anticancer effect of 10058-F4 combined with DOX can be replicated in clinical models. Accordingly, it is necessary to conduct more in-depth studies to determine the optimal dose of 10058-F4 in patients with TNBC and determine its safety profile.

In summary, TXNIP induced apoptosis and inhibited the growth of TNBC chemoresistant cells in vitro and in vivo via promoting ROS-dependent DNA damage. The small molecule c-Myc inhibitor 10058-F4 can promote TXNIP expression, increase intracellular ROS synthesis to decrease DOX-induced chemotherapy resistance in TNBC, and combined with DOX, enhance the cytotoxic effects of chemotherapeutic agents (Fig. 7N). These findings provide an experimental basis for applying TXNIP and 10058-F4 in the clinical treatment of drug-resistant TNBC.

## Supplementary information

Revised Manuscript - Marked Up

aj-checklist

Original Data File

Original Data File

## Data Availability

The datasets used during this study are available from the corresponding author on request.
