# Supplementary figures and images for: Identification of a novel mechanism for reversal of doxorubicin-induced chemotherapy resistance by TXNIP in triple-negative breast cancer via promoting reactive oxygen-mediated DNA damage
Source: Cell Death Dis. 2022 Apr 12;13(4):338. doi: 10.1038/s41419-022-04783-z (PMC9005717; doi:10.1038/s41419-022-04783-z)

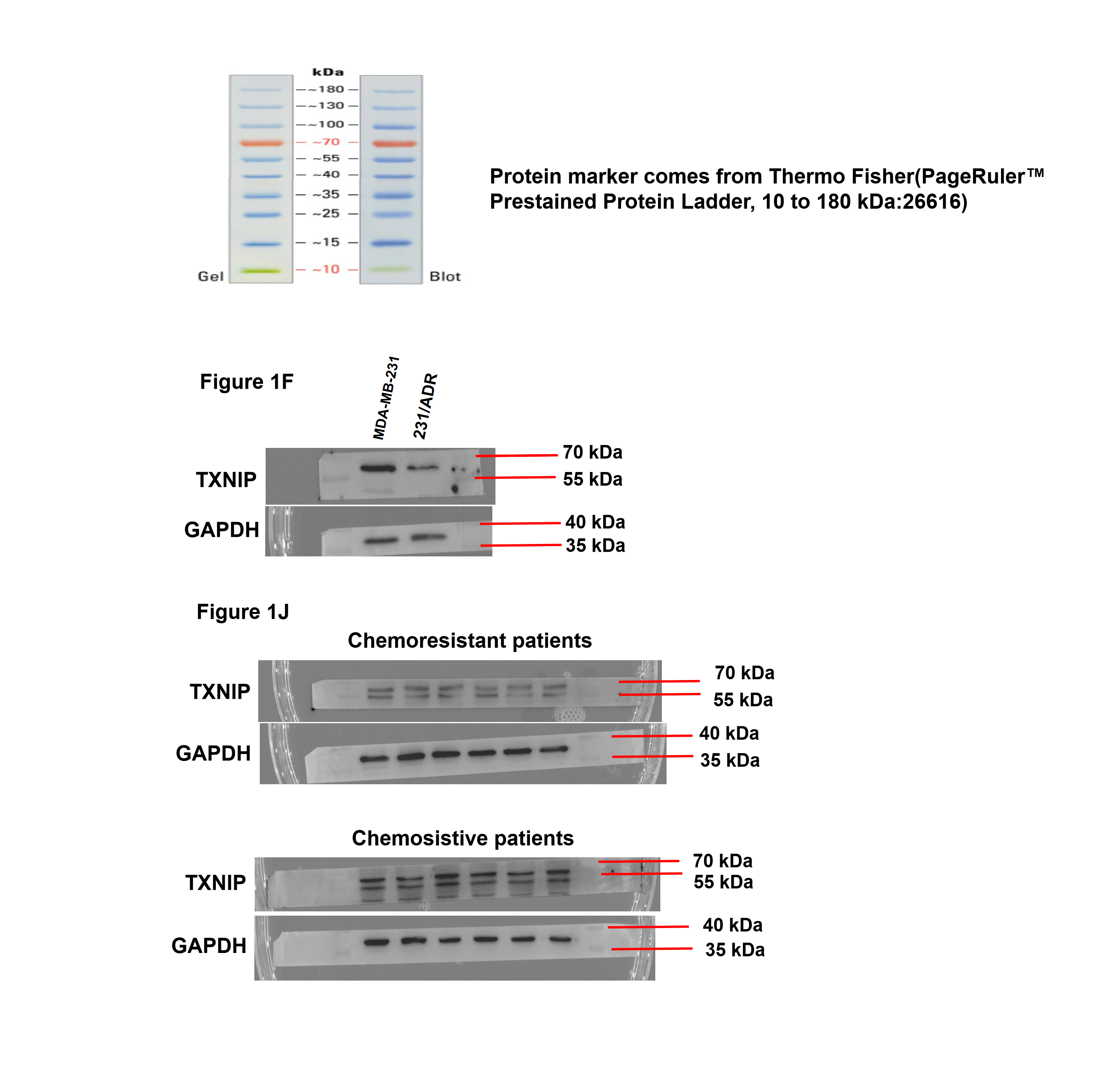

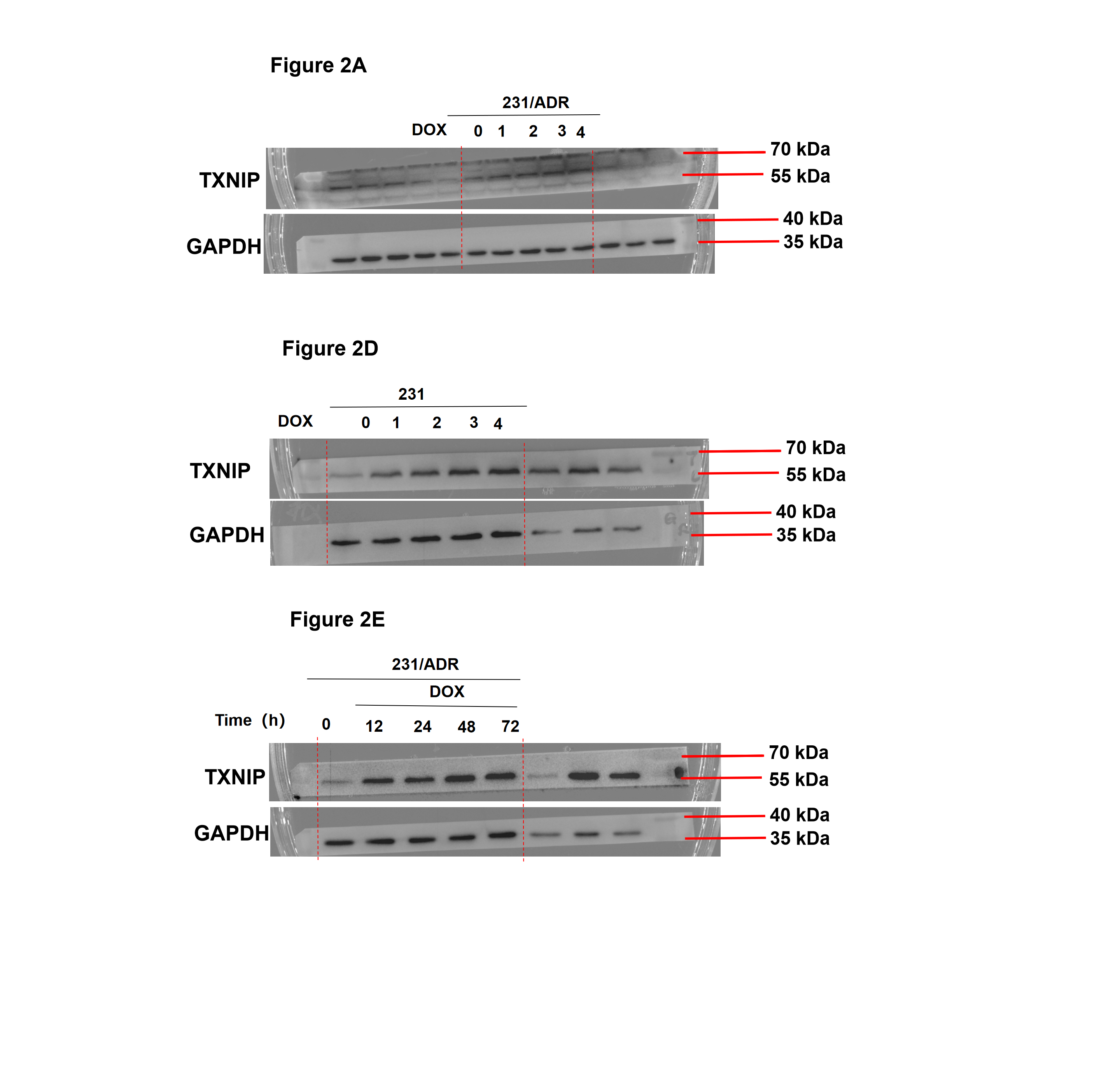


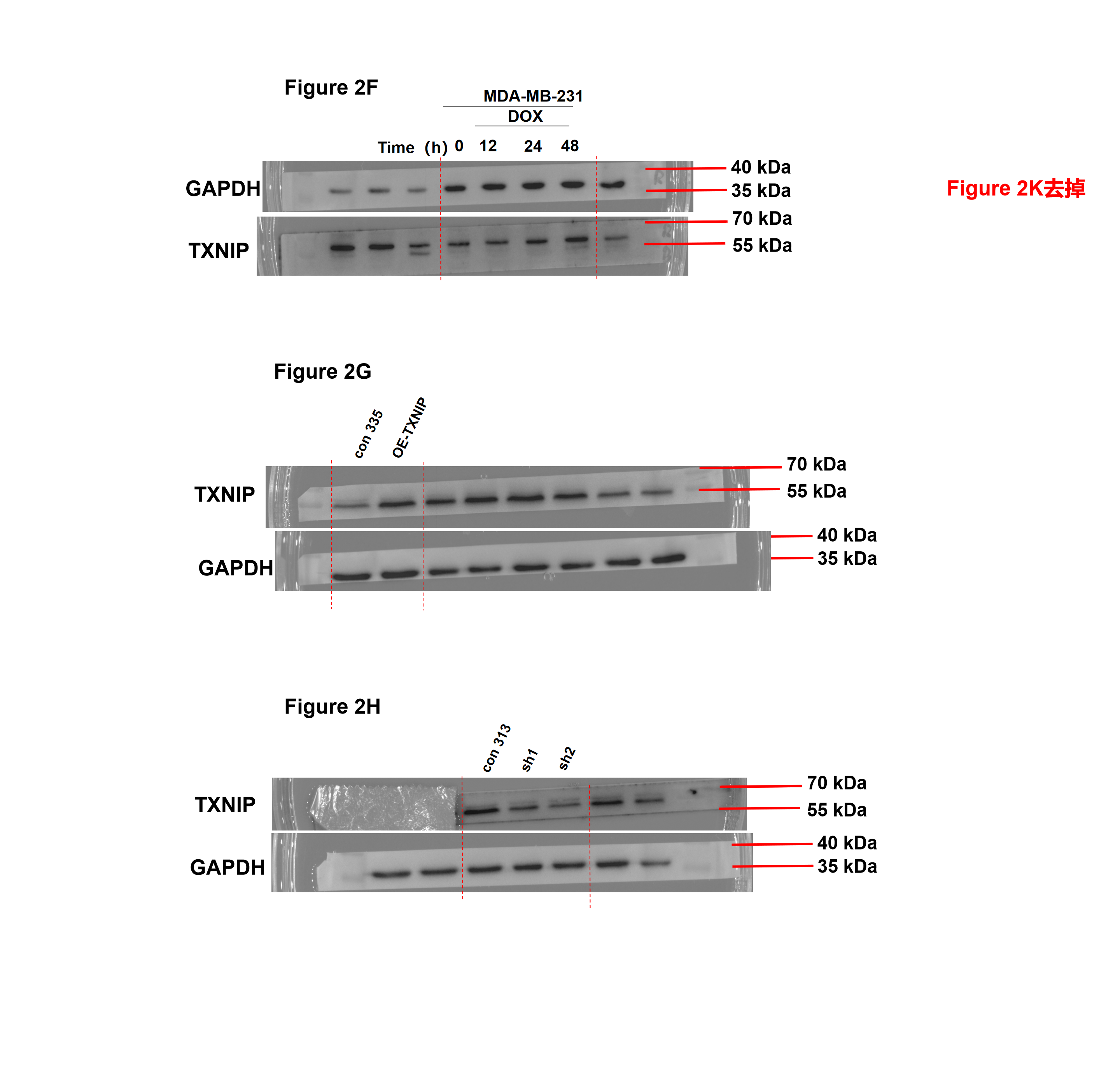

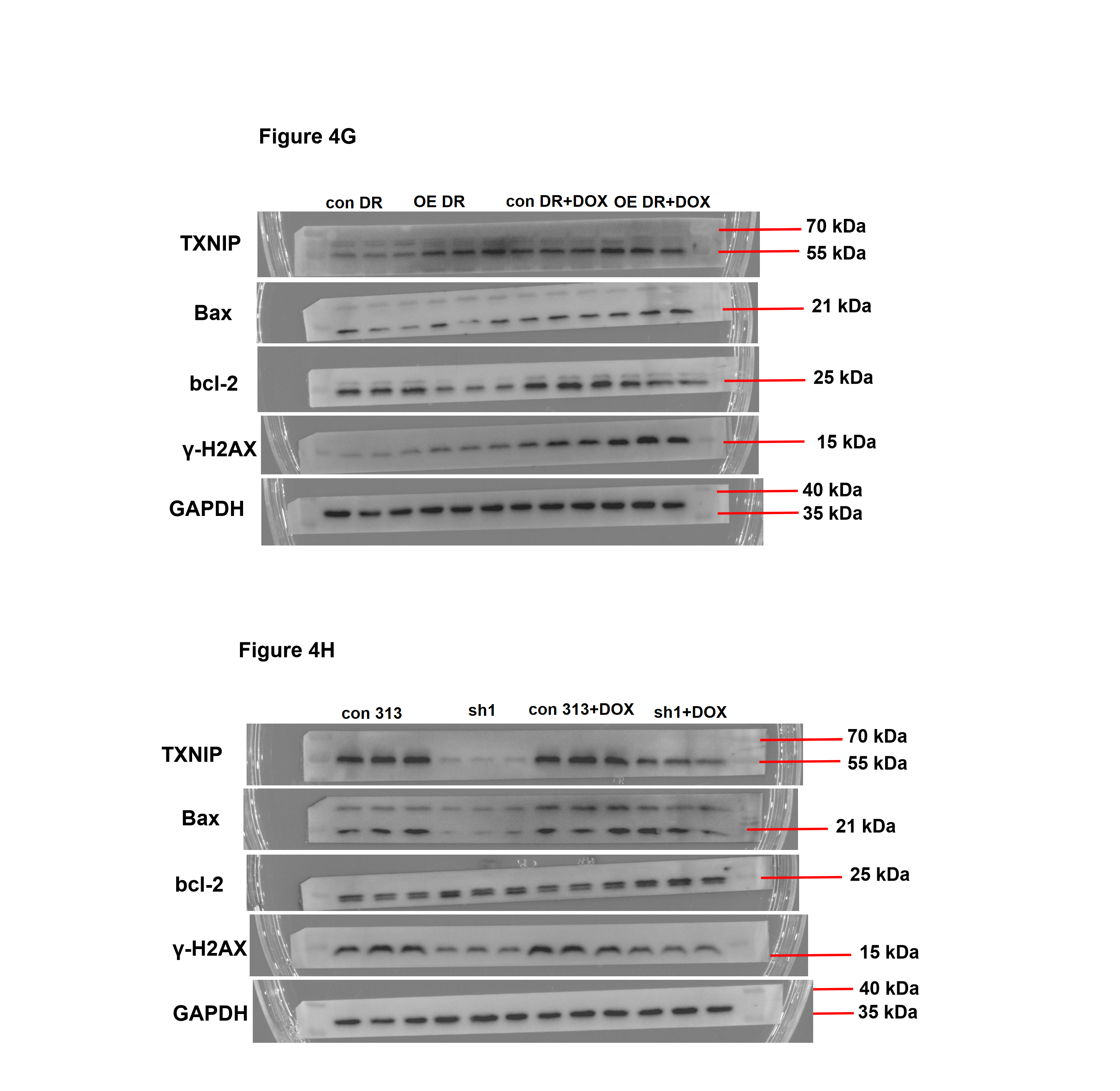


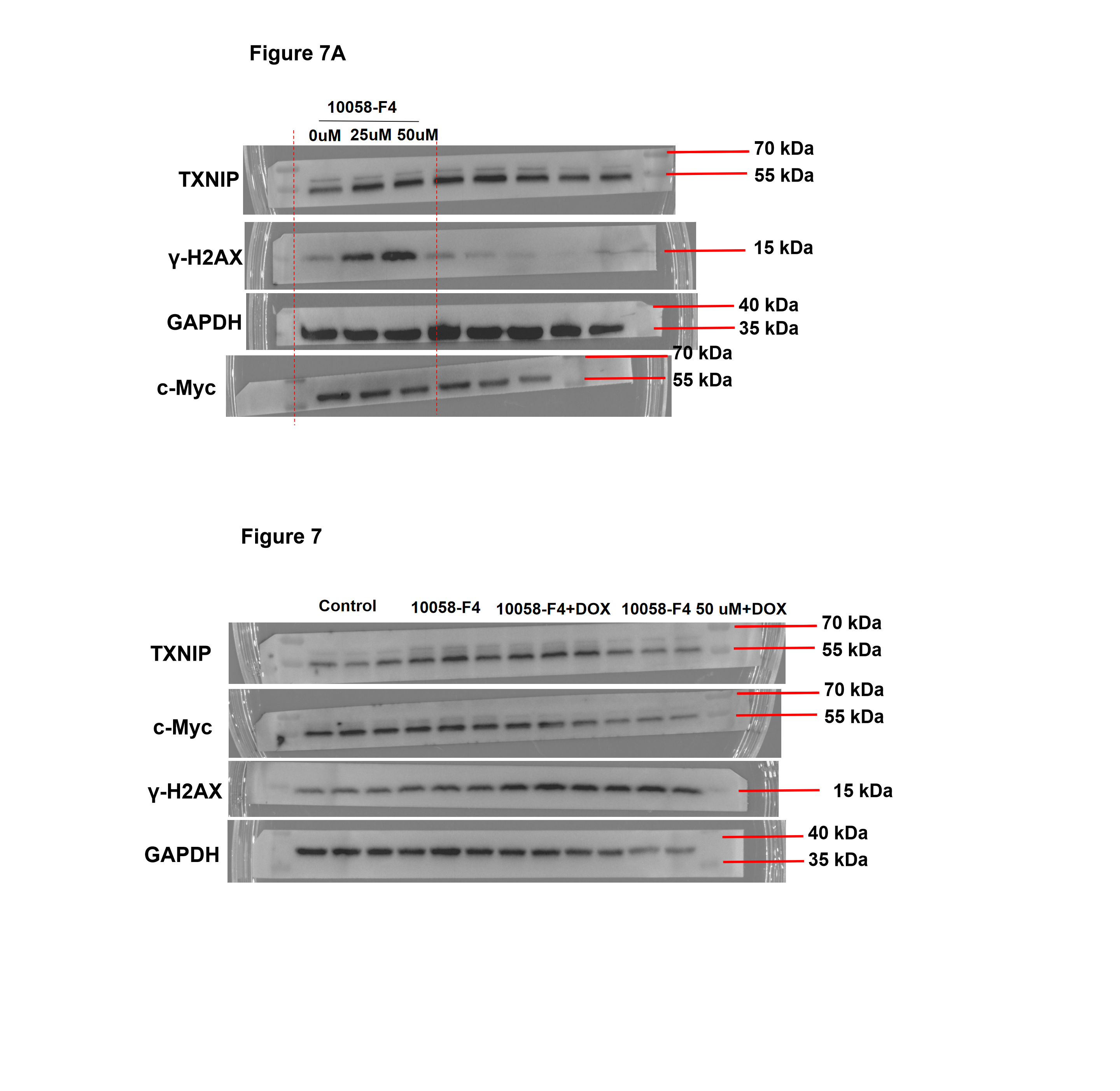


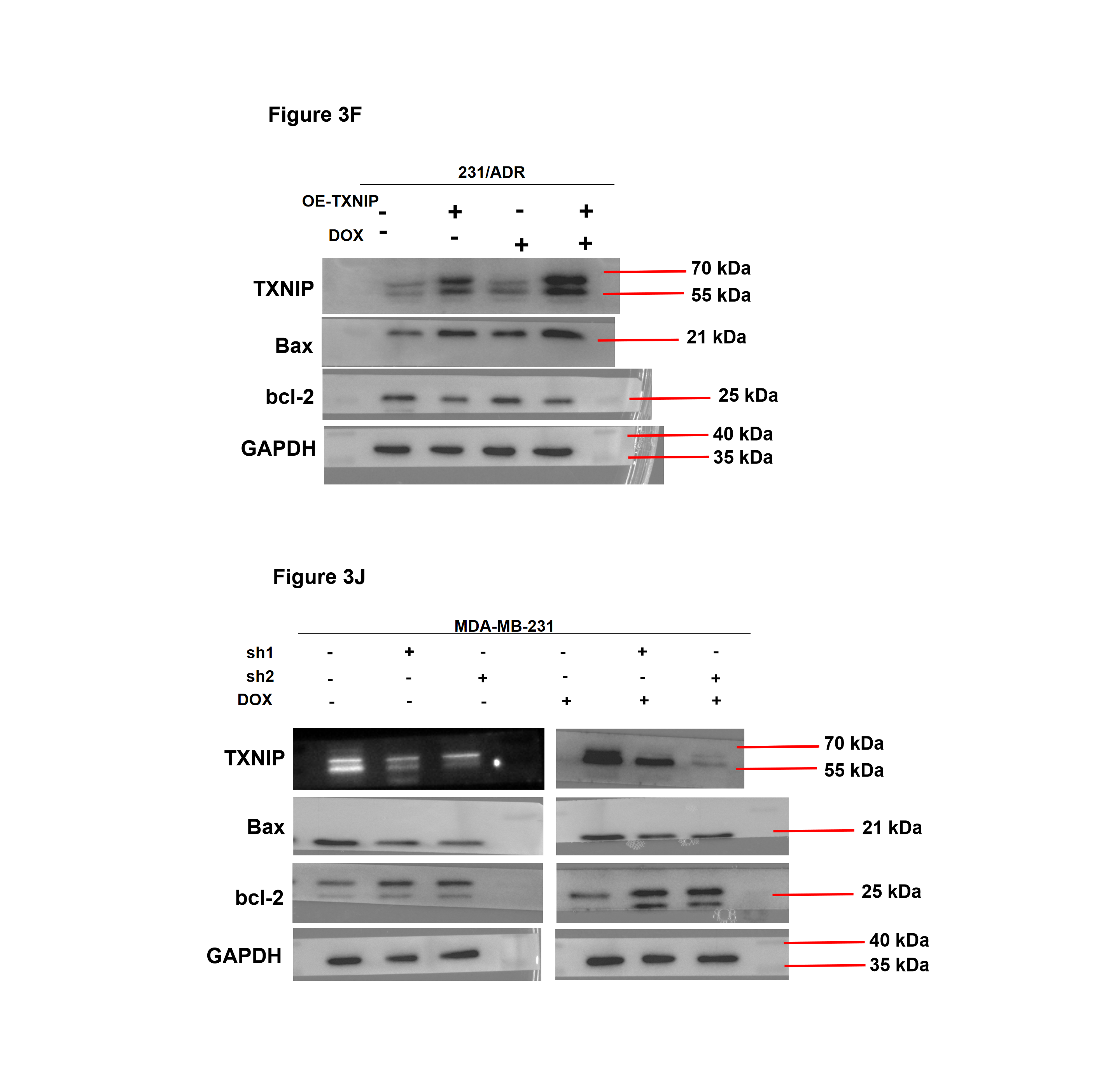


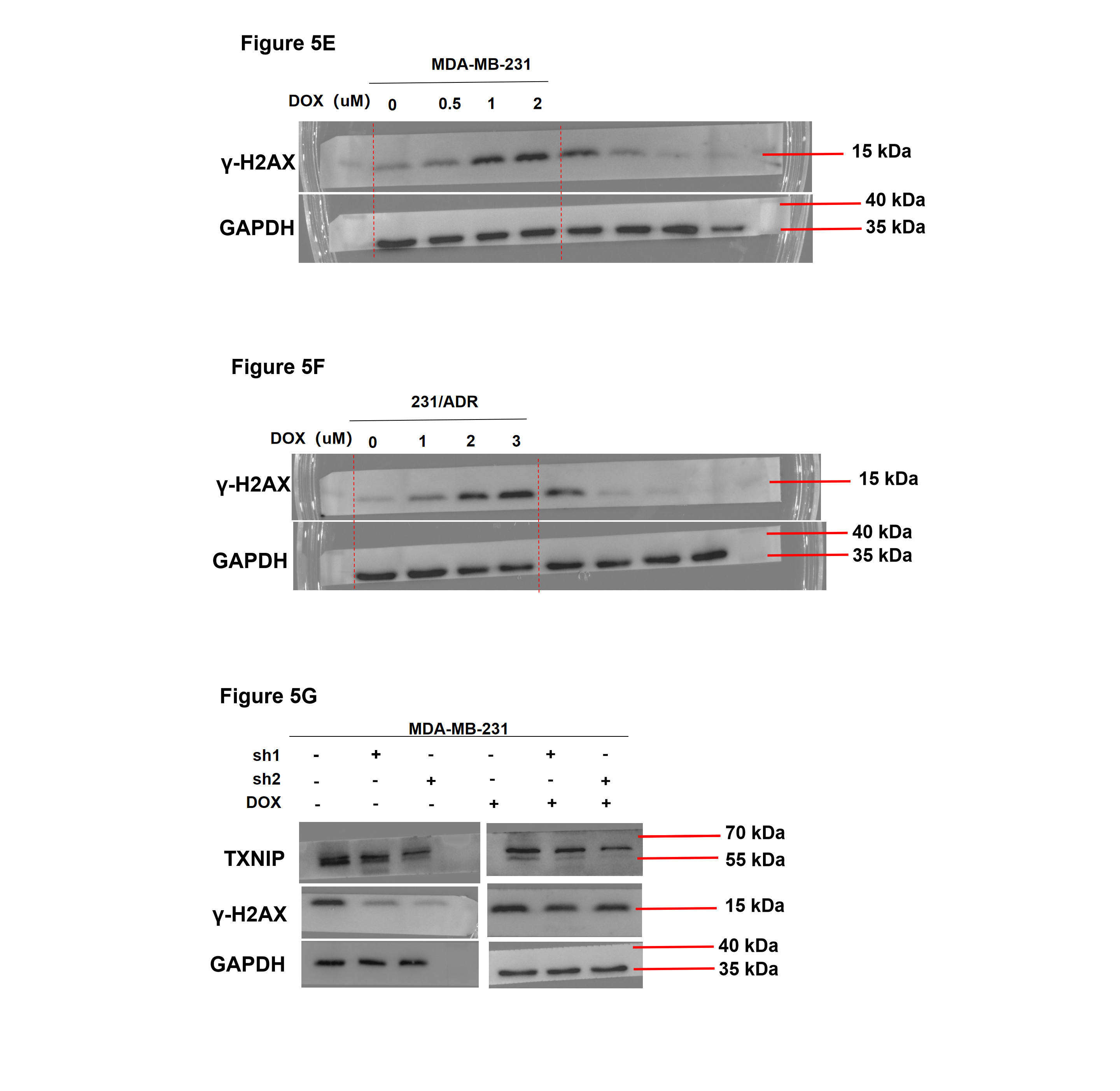

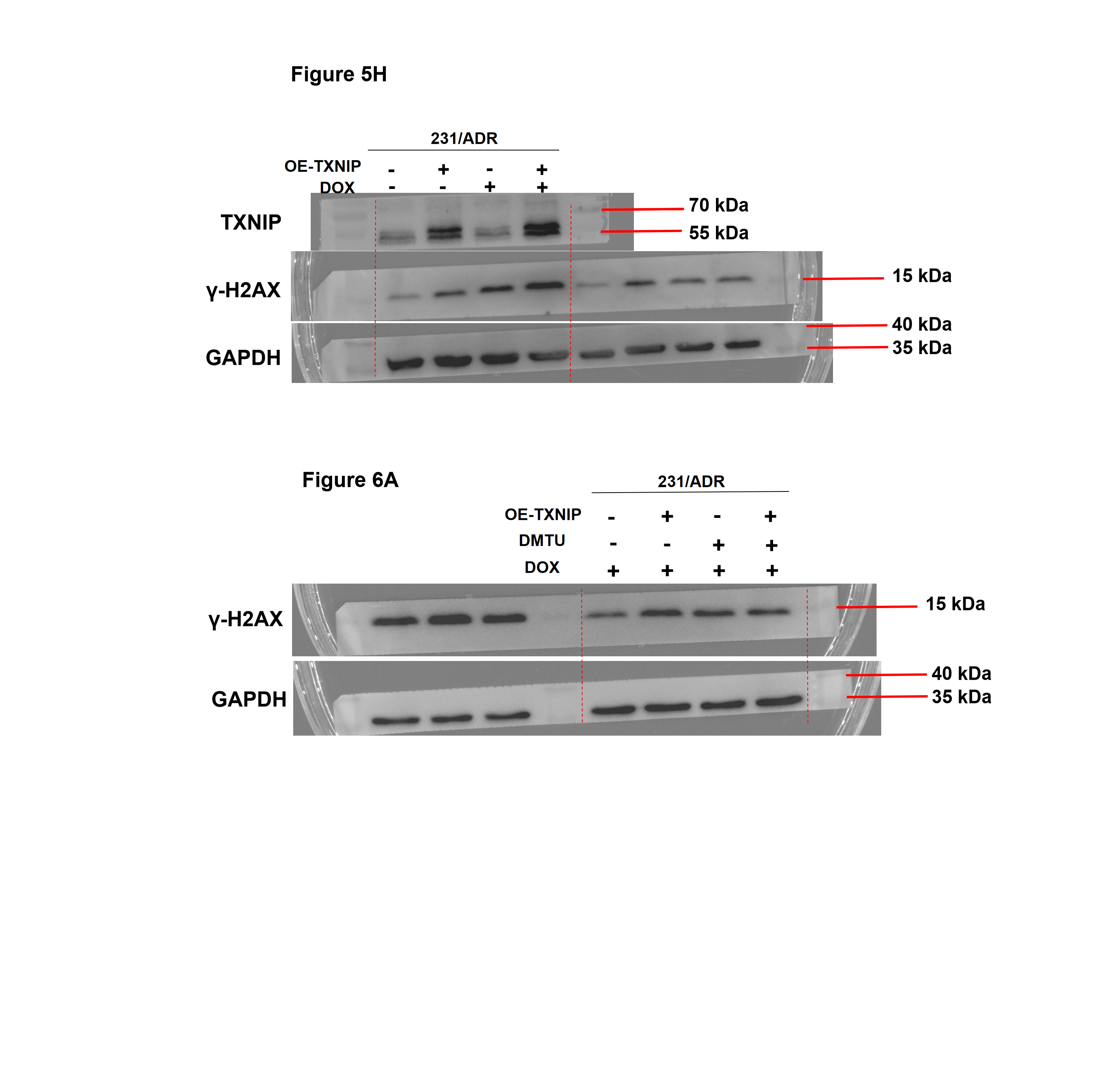

Supplement: Supplementary file 3 — Original Data File [file 41419_2022_4783_MOESM3_ESM.docx]
